# Angelman syndrome genotypes manifest varying degrees of clinical severity and developmental impairment

**DOI:** 10.1038/s41380-020-0858-6

**Published:** 2020-08-13

**Authors:** Marius Keute, Meghan T. Miller, Michelle L. Krishnan, Anjali Sadhwani, Stormy Chamberlain, Ronald L. Thibert, Wen-Hann Tan, Lynne M. Bird, Joerg F. Hipp

**Affiliations:** 1 Roche Pharma Research and Early Development, Neuroscience and Rare Diseases, Roche Innovation Center Basel, Basel, Switzerland; 2 Department of Neurology, Otto-von-Guericke-University, Magdeburg, Germany; 3 Department of Psychiatry, Boston Children’s Hospital, Boston, MA USA; 4 Harvard Medical School, Boston, MA USA; 5 Department of Genetics and Genome Sciences, University of Connecticut, Farmington, CT USA; 6 Department of Neurology, Massachusetts General Hospital, Boston, MA USA; 7 Division of Genetics and Genomics, Boston Children’s Hospital, Boston, MA USA; 8 Department of Pediatrics, University of California, San Diego, CA USA; 9 Department of Genetics/Dysmorphology, Rady Children’s Hospital, San Diego, CA USA

**Keywords:** Drug discovery, Autism spectrum disorders, Neuroscience, Genetics

## Abstract

Angelman Syndrome (AS) is a severe neurodevelopmental disorder due to impaired expression of *UBE3A* in neurons. There are several genetic mechanisms that impair *UBE3A* expression, but they differ in how neighboring genes on chromosome 15 at 15q11–q13 are affected. There is evidence that different genetic subtypes present with different clinical severity, but a systematic quantitative investigation is lacking. Here we analyze natural history data on a large sample of individuals with AS (*n* = 250, 848 assessments), including clinical scales that quantify development of motor, cognitive, and language skills (Bayley Scales of Infant Development, Third Edition; Preschool Language Scale, Fourth Edition), adaptive behavior (Vineland Adaptive Behavioral Scales, Second Edition), and AS-specific symptoms (AS Clinical Severity Scale). We found that clinical severity, as captured by these scales, differs between genetic subtypes: individuals with *UBE3A* pathogenic variants and imprinting defects (IPD) are less affected than individuals with uniparental paternal disomy (UPD); of those with *UBE3A* pathogenic variants, individuals with truncating mutations are more impaired than those with missense mutations. Individuals with a deletion that encompasses *UBE3A* and other genes are most impaired, but in contrast to previous work, we found little evidence for an influence of deletion length (class I vs. II) on severity of manifestations. The results of this systematic analysis highlight the relevance of genomic regions beyond *UBE3A* as contributing factors in the AS phenotype, and provide important information for the development of new therapies for AS. More generally, this work exemplifies how increasing genetic irregularities are reflected in clinical severity.

## Introduction

Angelman syndrome (AS) is a rare genetic neurodevelopmental disorder with a prevalence of 1 in 10,000–24,000 births [1, 2]. Clinical characteristics of AS include global developmental delay, intellectual disability, epilepsy, and sleep difficulties [3–6].

AS is due to the lack of expression of the maternal copy of *UBE3A* in the chromosome 15q11–13 region [6, 7]. In healthy individuals, the paternal copy of *UBE3A* is silenced in neurons by genomic imprinting [8]. In AS, *UBE3A* expression is impaired either through deletions including the maternal copy of *UBE3A* or through one of several other mechanisms: pathogenic variants of the maternal copy of *UBE3A* (Mut), imprinting defects (IPD), and paternal uniparental disomy (UPD) of chromosome 15 [9]. Deletions account for ~70% of all AS diagnoses, *UBE3A* pathogenic variants, IPD, and UPD for ~10% each [6]. Some patients with AS-like symptomatology have no or unclear genetic abnormalities [6, 7, 9, 10] and are not investigated here.

## AS subtypes with different genetic mechanisms (Fig. 1)

Among individuals with a deletion, the length of the chromosomal deletion varies. Deletions of 15q11–q13 commonly occur at recurring breakpoints, resulting in two typical deletion sizes: class 1 (Del1, ~6 Mb, ~16 genes, and various noncoding regions deleted, ~40% of deletions) and class 2 (Del2, ~5 Mb, ~12 genes, and various noncoding regions deleted, ~55% of deletions). Atypical deletions (DelAT, ~5%) can span chromosomal segments longer than Del1 or shorter than Del2 [11, 12].

**Fig. 1:**
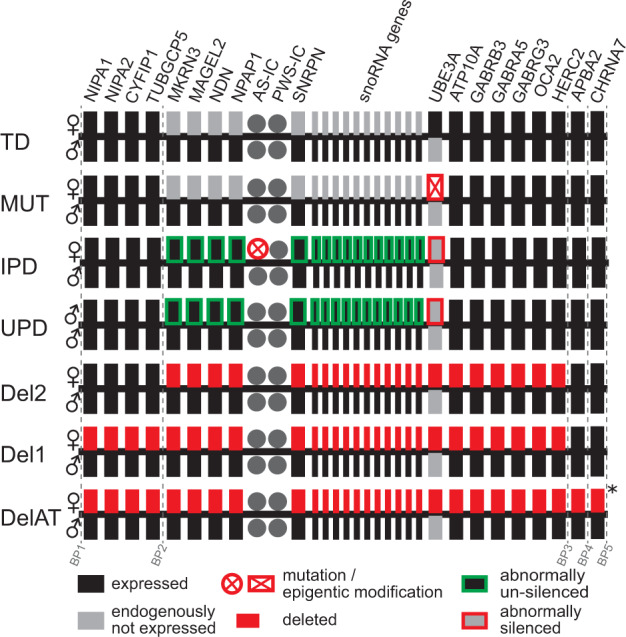
Schematic representation of different AS genotypes in chromosome 15q11–13. ♀ Maternal, ♂ Paternal. TD typically developing, mono-allelic expression of some genes is controlled by genomic imprinting. These genes are “endogenously not expressed”. Mut: UBE3A pathogenic variants, can be truncating or missense mutations. IPD imprinting center defects. Maternal UBE3A is not expressed due to impairments in the imprinting process, some of which have a deletion of the imprinting center (AS-IC). IPD goes in hand with overexpression of paternally expressed genes (MKRN3–SNRPN). Some individuals have mosaicism, i.e., the IPD affects only a subset of cells. UPD paternal uniparental disomy. Paternal gene expression from both copies leads to the lack of expression of UBE3A and overexpression of paternally expressed genes (MKRN3–SNRPN). IPD and UPD should be identical in their consequences. Del1/Del2 deletion class 1 and 2 with characteristic breakpoints; DelAT atypical deletion. Can be shorter or longer than Del1 and Del2. An asterisk indicates additional deleted genes of varying length (could also be less than for Del1/Del2).

Individuals with UPD have two paternal copies of the chromosome 15q11–q13 segment and therefore two silenced copies of *UBE3A*, resulting in a near-complete lack of expression in neurons. Furthermore, genes and noncoding sequences in this region that are imprinted and paternally expressed are likely overexpressed as there are two active copies in UPD patients [13].

Imprinting center defects (IPDs) can result from epigenetic events (~85%) or deletions within the AS imprinting center (~15%) and effectively cause the maternal chromosome 15q11q13 region to “behave” like the paternal copy. Therefore, IPD can be expected to be effectively like UPD [14–16]. However, a substantial fraction (~30%) of individuals with IPD exhibit mosaicism (i.e., genetic defect only in a subset of cells) [17].

*UBE3A* pathogenic variants lead to a selective impairment of expression of functional UBE3A protein, leaving expression of other genes presumably intact [18]. Many of these variants occur de novo, but a substantial portion are inherited from a mother who carries the mutation on her paternally inherited gene [9]. *UBE3A* pathogenic variants can be further grouped into missense mutations (MutM) and truncating mutations (MutT). Whereas truncating mutations highly likely lead to a complete lack of *UBE3A* expression, missense mutations may lead to production of a modified UBE3A protein that retains residual functionality [19, 20].

## Differences in clinical features and disease severity between AS genotypes

To our knowledge, nine previous studies have characterized the developmental and clinical differences between AS genotypes (summarized in Supplementary Table 1). Taken together, these studies consistently show a more severe clinical phenotype for AS individuals with a deletion compared with those without a deletion, and some suggest that larger deletions lead to more severe impairment than smaller deletions. Possible differences between non-deletion subtypes (MutM, MutT, IPD, UPD) are inconsistent or have not been investigated. Previous studies had limited sample sizes, compared only a subset of the different genotypes, or focused on a limited set of symptoms; therefore, a comprehensive analysis of the relationships between genotype and clinical features in AS across a broad spectrum of clinical and performance measures is needed.

Using a statistical modeling approach and the largest sample of individuals with AS studied so far, we systematically investigated differences between AS genotypes for several cognitive and developmental domains, with a focus on standardized psychometric developmental tests and questionnaires.

## Patients and methods

See the Supplementary Patients and Methods for an extended description.

The reported data were obtained as part of the AS Natural History Study (ASNHS) (ClinicalTrials.gov Identifier: NCT00296764), a longitudinal multicenter study of AS. A subset of these data have been analyzed previously [21]. Consent was obtained according to the Declaration of Helsinki and was approved by the institutional review boards of the participating sites.

### Participants

Per study protocol, participants were seen approximately annually over 8 years (mean number visits: 2.9). Data reported here are from 250 participants (848 datasets; 127 females) that fall into one of six genetic subgroups (MutM, MutT, IPD, UPD, Del1, Del2; see Supplementary Table 2) in the age range 1–18 years. Mean age at clinic visits was 82.4 ± 45.3 months (Supplementary Fig. 1).

### Clinical scales

We analyzed data from the Bayley Scales of Infant Development, Third edition (BSID-III) [22], the Vineland Adaptive Behavior Scales, Second edition (VABS-2) [23, 24], the Preschool Language Scale, Fourth edition (PSL-4) [25] (all distributed by Pearson Education Inc., London, www.pearsonclinical.com), and the Clinical Severity Scale (CSS), a scale developed for the ASNHS. Trained personnel (physicians and licensed psychologists) carried out all assessments (for number of datapoints for each scales see Supplementary Table 3). The CSS has not been published previously. A detailed description of the CSS can be found in the Supplementary Table 4. In brief, the CSS encompasses 11 items across five domains: seizures, growth, motor abilities, scoliosis, language, and global development. The study protocol and tests performed were identical across all sites.

### Data analysis

**Fig. 2:**
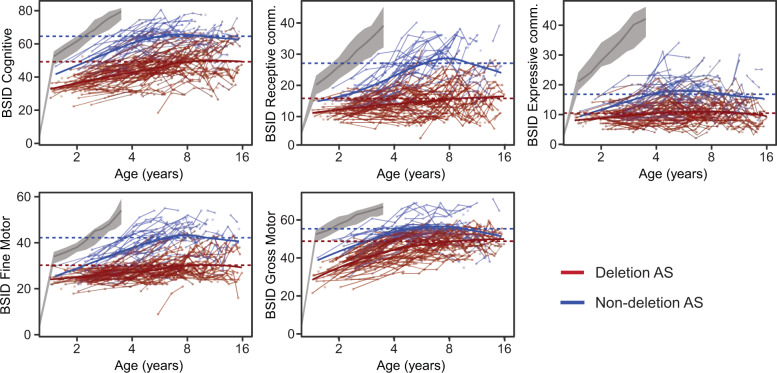
Raw data from the five BSID-III scales as a function of age. Gray bands indicate median scores and inter-quartile ranges from a typically developing sample (data from the scale manuals, available for up to 3.5 years of age). Values from the same participant are connected by thin lines. Thick lines are the LOESS smoothing curves for deletion (blue) and non-deletion (red) participants. Dotted horizontal lines are the overall group means. Note that these curves are cross-sectional data summaries, i.e., they do not account for within-subject longitudinal effects and are used for qualitative inspection of the developmental trajectory.

Data were analyzed using linear mixed-effects models (LMM). We fit a LMM to the raw scores of each subscale and the CSS sum score. We modeled random intercepts per participant (to account for repeated measurements) and per study site (random intercept for each of the six centers of the study, to capture possible experimenter-induced covariance between participants seen at the same site). As fixed effects, we specified a third-order mean-centered orthogonal polylogarithmic function of age. We chose this parameterization to capture nonlinear developmental trajectories apparent from visual inspection of the data (see Fig. 2, Supplementary Figs. 2 and 3).

First, we tested for differences between participants with (Del1, Del2) and without (MutT, MutM, IPD, UPD) deletions. For each scale, we compared a model using only age but no genotype information to a model with additional information about the presence or absence of a deletion and the interaction of the presence or absence of a deletion with age. We then separated the dataset into deletion and non-deletion participants and further compared subgroups within them. We tested whether introducing diagnostic information concerning the class of deletion (Del1, Del2) and subtype of non-deletion (MutM, MutT, IPD, UPD) would significantly improve the models using likelihood-ratio tests (LRT).

When the best model contained the full diagnostic information for the non-deletion group, we performed pair-wise post-hoc comparisons between genotypes. We adjusted the *p* values obtained in these post-hoc comparisons using the Benjamini–Hochberg method [26].

**Fig. 3:**
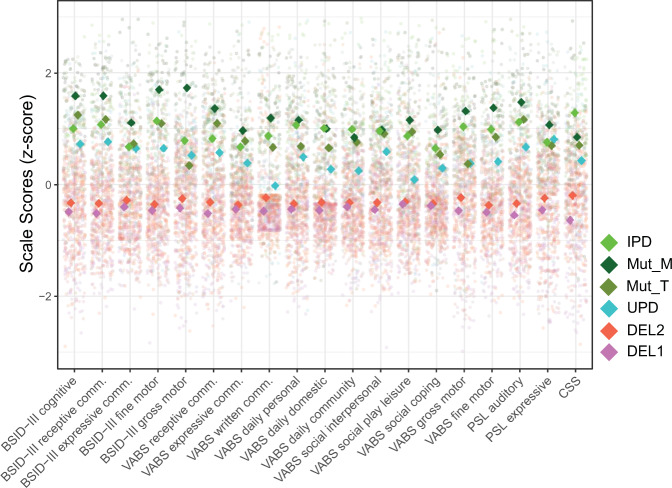
Comparisons of scores for different genotypes for mean log age. *Z*-standardized data from all participants and visits with the; data projected to mean of log_2_ age (5.8 years), derived from the respective “winning model” (see Patients and Methods). See Supplementary Fig. 4 for mean ± 1 SD around the mean log age (i.e., 3.2 and 10.7 years, respectively). This Fig. (and Supplementary Fig. 4) serves illustrative purposes and indicates the directionality of effects, statistical analyses are provided in Tables 1,2, and Supplementary Tables 5, 6, 10. CSS values have been inverted, such that higher values reflect higher performance.

We used the coefficients of the “best model” for each scale (i.e., the level of genotype detail as found in the analyses reported in Supplementary Tables 5 and 6, and Table 1) to predict values at the sample mean ± standard deviation (std) of log age (3.2, 5.8, 10.7 years) to generate a summarizing visualization of genotype differences (reported in Fig. 3, Supplementary Fig. 4). Furthermore, to investigate possible structure in the inter-individual variability across scales, we performed a factor analysis.

**Table 1:** Model comparisons within the non-deletion subgroup.

Scale	*χ*^*2*^	*p*	MutT fixed eff	MutM fixed eff	UPD fixed eff	*F*_*MAIN*_	Domain
BSID-III cognitive	24.57	**0.03**	1.65	2.67	−3.99	3.82	Cognitive
BSID-III receptive comm.	23.45	**0.04**	−0.48	0.73	−2.7	2.06	Communication
BSID-III expressive comm.	15.96	0.2	0.58	1.1	0.39	0.33	Communication
BSID-III fine motor	18.97	0.11	−1.3	1.24	−4.32	3.67	Motor
BSID-III gross motor	34.07	**<0.001**	−3.09	3.35	−2.43	4.16	Motor
VABS receptive comm.	24.3	**0.03**	0.66	2.35	−1.58	2.67	Communication
VABS expressive comm.	22.42	**0.04**	−3.03	−1.68	−3.92	2.83	Communication
VABS written comm.	34.62	**<0.001**	−1.26	−0.09	−2.41	6.02	Communication
VABS daily personal	21.85	0.05	−5.91	−3.41	−6.14	2.35	Daily living
VABS daily domestic	53.82	**<0.001**	−2.95	−2.25	−4.44	5.81	Daily living
VABS daily community	31.64	**0.01**	−1.47	−1.33	−2.94	3.15	Daily living
VABS social interpersonal	25.44	**0.02**	−0.85	−1.26	−2.11	1.67	Social
VABS social play leisure	29.43	**0.01**	−1.96	−0.83	−6.47	4.97	Social
VABS social coping	9.85	0.63	−2.27	−1.11	−2.15	1.51	Social
VABS gross motor	28.96	**0.01**	−9.53	0.34	−8.38	7.01	Motor
VABS fine motor	30.29	**0.01**	−3.33	−0.37	−4.6	3.53	Motor
PSL auditory	35.41	**<0.001**	−0.57	−0.05	−2.73	2.70	Communication
PSL expressive	40.84	**<0.001**	−0.44	0.84	0.16	0.42	Communication
CSS	27.24	**0.02**	−3.28	−3.04	−3.76	4.10	Clinical

*F* values for main effect of deletion length. *P* values have been obtained in likelihood-ratio tests (df = 11) and corrected for multiple comparisons using FDR (significant *p*-values, i.e. *p* < 0.05, are shown in bold font).. Unadjusted *p* values can be found in the Supplementary Table 9. The ‘Mut fixed effect’ column indexes the fixed main effect of Mut, the ‘UPD fixed effect’ indexes the fixed main effect of UPD, both as estimated by the LMM models, compared with IPD, and in units of raw points.

## Results

We analyzed 848 datasets from 250 individuals with AS (127 females, i.e., 50.8%). Visual inspection of the BSID-III scales (Fig. 2, Supplementary Fig. 2), and similarly VABS-2, PLS-4, and CSS scales (Supplementary Fig. 3) suggest a nonlinear developmental trajectory with a steady increase and then plateau at about age 6 years. We accounted for this developmental trajectory using 3rd order polynomials of log age in subsequent analyses (see Patients and Methods). Overall, test results were relatively stable within individuals across time (ICC: 0.62 ± 0.090, min 0.42, max 0.79; see Supplementary Table 7).

### Differences in clinical features between deletion and non-deletion AS

First, we compared the scores on the BSID-III scales between deletion and non-deletion AS. A model differentiating the deletion and non-deletion genotypes fit the data significantly better (compared with a simple model without differentiation), with higher scores for individuals with non-deletion AS for all five domains (LRT, *χ*^*2*^ > 100, *p* < 0.001 for all scales, see Supplementary Table 5; see Fig. 3 and Supplementary Fig. 4).

These results may have been biased by participants for whom the study physician or psychologist decided to skip the BSID-III assessment due to expected or observed ceiling effects. Indeed, the proportion of individuals without BSID-III assessments significantly differed between groups with 25.5% for individuals with non-deletion AS and only 0.6% for individuals with deletion AS (Chi-square test comparing difference in frequencies, *p* = 5.6 × 10^−11^; Supplementary Table 8; Supplementary Fig. 5).

In line with the BSID-III, all domain scores from all other scales investigated (VABS-2, PLS-4, CSS) showed significantly higher scores for non-deletion compared with deletion AS (LRT, *χ*^*2*^ > 82, *p* < 0.001 for all scales; see Supplementary Table 5). Notably, several domains of the VABS-2 showed flooring effects, while the PLS-4 and the CSS were psychometrically as “well-behaved” as the BSID-III (no major flooring effects, coverage of a substantial fraction of possible values across age and genotype; see Supplementary Fig. 3). In sum, our results confirmed prior evidence that individuals with deletion AS generally have a more severe neurodevelopmental phenotype than individuals with non-deletion AS.

Next, we investigated differences in clinical presentation within deletion and non-deletion subgroups, respectively.

### Dependence of clinical features on deletion size

A model differentiating deletion classes 1 and 2 fit the data significantly better compared with a simple model without differentiation for only 1 out of 19 scales tested, the CSS (*p* < 0.05; FDR corrected; LRT; Supplementary Table 6). However, numerically, for all scales, individuals with Del1 scored lower than those with Del2 (Fig. 3). In sum, our results suggest only minor differences in clinical severity as measured by CCS between the common deletion genotypes.

### Clinical features of non-deletion AS depend on specific genotype

A model differentiating the four non-deletion subtypes (UPD, IPD, MutM, MutT) fit the data significantly better compared with a simple model without differentiation for 15 out of 19 scales tested (log-LRT; *p* < 0.05; FDR corrected; Table 1, Supplementary Table 9).

To illustrate differences in clinical features across non-deletion genotypes, we projected all data to the mean age (given the model splitting the non-deletion genotypes, Fig. 3). Numerically, individuals with UPD scored on average lower than all other non-deletion genotypes and MutM scored higher than all other non-deletion genotypes, while MutT and IPD scored in between on most scales. Age projections for younger (3.2 years) and older (10.7) ages (mean log age ± 1 SD; Supplementary Fig. 4) suggest that genotype differences tend to increase with age.

In line with these qualitative observations, post-hoc statistical comparisons (for the 15 scores found significant above) revealed that for many scales, UPD indeed scored significantly lower compared with all other non-deletion genotypes (UPD compared with MutM: 7/15, MutT: 4/15, IPD: 12/15; *p* < 0.05; FDR corrected) and MutM tended to score significantly higher (IPD: 1/15 contrasts significant, UPD: 7/15, MutT: 5/15), see Table 2.

**Table 2:** Pair-wise post-hoc comparisons for non-deletion genotypes.

Scale	IPD vs. UPD	MutT vs. UPD	MutM vs. UPD	MutM vs. MutT	IPD vs. MutT	IPD vs. MutM	Domain
BSID-III cognitive	**0.046**	**0.043**	0.085	0.769	0.13	0.301	Cognitive
BSID-III receptive comm.	**0.028**	0.074	**0.02**	0.416	0.906	0.955	Communication
BSID-III gross motor	0.171	0.065	**0.006**	**0.005**	0.396	**0.016**	Motor
VABS receptive comm.	**0.031**	0.085	0.075	0.182	0.619	0.218	Communication
VABS expressive comm.	0.052	0.718	0.416	**0.011**	0.462	0.075	Communication
VABS written comm.	**0.012**	0.096	**<0.001**	**0.034**	0.651	0.292	Communication
VABS daily domestic	**<0.001**	**0.035**	**<0.001**	**0.007**	0.421	**0.028**	Daily living
VABS daily community	**0.001**	0.067	0.209	0.72	0.265	0.612	Daily living
VABS social interpersonal	**0.047**	0.214	0.143	0.784	0.918	0.202	Social
VABS social play leisure	**0.007**	0.052	**0.031**	0.784	0.976	0.583	Social
VABS gross motor	**0.046**	0.265	**0.028**	0.052	0.101	0.16	Motor
VABS fine motor	**0.035**	**0.003**	**0.011**	0.26	0.265	0.973	Motor
PSL auditory	**0.023**	**<0.001**	0.052	0.16	0.26	0.927	Communication
PSL expressive	**0.052**	**<0.001**	0.301	**<0.001**	0.214	0.065	Communication
CSS	**0.005**	0.218	0.271	0.976	0.069	0.155	Clinical

*P* values have been obtained through model-comparing likelihood-ratio tests and are corrected for multiple comparisons using FDR. Unadjusted *p* values can be found in the Supplementary Table 9. The directionality of the effects can be inferred from Table 1 (fixed effects) and Fig. 3.

Bold values indicate significant *p*-values, i.e. *p* < 0.05.

Our results revealed that individuals with UPD are more severely impaired than other non-deletion types and, in particular, more impaired than MutT, the genetic group that highly likely leads to a specific and complete impairment of *UBE3A* expression. This raises the question of whether UPD would be phenotypically closer to deletion AS compared with other non-deletion AS genotypes. We therefore compared UPD with DEL2, the shorter deletion genotype. UPD has indeed higher scores compared with DEL2 (the shorter and less impaired deletion) for 17/19 scales (*p* < 0.05; Benjamini–Hochberg corrected, FDR = 0.05, Supplementary Table 10).

In sum, our results suggest an ordered phenotypic impairment of MutT < UPD < Del2, where UPD is in between MutT and Del2 in terms of clinical severity as assessed by the 19 scales.

### Functional domains

The differences between deletion and non-deletion genotypes as well as between different non-deletion genotypes spanned all functional domains captured by the scales including cognitive, social, communication, daily living skills, and motor domains (see Supplementary Table 10 and Table 2). Thus, the identified genotype differences reflect a “global factor”, rather than domain-specific, developmental, and clinical differences.

Motivated by these results, we asked if the clinical scales used here are able to differentiate functional domains in AS. To this end, we investigated the correlation structure between all 19 scales and performed a factor analysis (see Supplementary Fig. 6, Supplementary Table 11). Most scales showed moderate to high correlations between similar domains across different scales, and the factor analysis revealed a plausible factor structure, where measures from the same domain (e.g., scales capturing motor symptoms or communication, respectively) load on the same factors. This indicates that the scales can meaningfully and consistently capture different functional domains.

## Discussion

Using the largest clinical dataset to date, we confirm previous evidence and clinical intuition that individuals with deletion AS are more impaired than non-deletion AS. We then revealed differences in clinical features within non-deletion AS genotypes.

### Deletion AS

We corroborated previous findings that individuals with deletion AS are on average clinically and developmentally more severely impaired than individuals with non-deletion AS. This is genetically plausible because deletions include additional genes that likely have an impact on development and brain function. Genes that may drive the difference include three GABA_A_ receptor subunit genes (*GABRB3*, *GABRG3*, *GABRA5*) that are single-copy (haploid) for the deletion genotypes and intact (diploid) for all non-deletion genotypes. Indeed, loss of function variants in these GABA_A_ subunit genes have been linked to epilepsy and developmental delay [27]. In line with these results, recent electrophysiological evidence suggests that differences in brain rhythms between deletion and non-deletion AS may relate to altered GABAergic signaling [28].

There was prior evidence that individuals with class 1 (larger) deletions might be more impaired than individuals with class 2 deletions in the domains of language, cognition and motor [11, 29]. Furthermore, individuals with deletions of only the genes that are additionally deleted in class 1 compared with class 2 AS often present with developmental delay and psychiatric syndrome (15q11.2 BP1–BP2 Microdeletion Syndrome [30],) suggesting an role of these genes in neurodevelopment. We could confirm significant differences between deletion subtypes only for the CSS, but not for the other scales. Numerically, all 19 scales tested of the VABS-2, BSID-III, and PLS-4 were lower for individuals with class 1 deletions suggesting that, with an increasing sample size, other domains may reach statistical significance. This suggests that, from a practical perspective, differences between deletion classes are small. However, relevant differences between these deletion AS subgroups may not be captured with the clinical scales analyzed herein.

### Non-deletion AS

Non-deletion AS genotypes have a low prevalence (~10% of 1 in 10,000–24,000 for IPD, UPD, and Mut, respectively) such that even specialized clinicians see only a few patients from each non-deletion subtype in their professional lives. The order of clinical severity within the non-deletion AS population had previously not been systematically examined. The current study addressed this question by using a large dataset, collected across six expert centers over ~8 years allowing investigation of differences in clinical features across non-deletion AS subgroups.

The results revealed that individuals with UPD have lower scores on the investigated clinical scales compared with individuals with other non-deletion genotypes (MutM, MutT, IPD). In particular, individuals with UPD exhibit lower scores compared with individuals with truncating variants (MutT), the genetic subgroup that highly likely leads to a specific and complete UBE3A disruption in neurons. Mouse models of AS suggest no relevant quantity of Ube3a postnatally [31]. However, it has previously been hypothesized that imprinting of *UBE3A* is ‘leaky’, i.e., not leading to a 100% silencing, such that *UBE3A* in UPD could have additional residual expression from two incompletely silenced copies compared with the residual expression from one incompletely silenced copy in MutT, which may be of functional relevance (Arthur Beaudet, personal communication). If the ‘leaking hypothesis’ were true, UPD should be less affected than MutT (complete disruption of maternal *UBE3A* expression and ‘leaky’ expression from only one copy), but we found the opposite. Our results suggest that, if existent, a residual expression of silenced *UBE3A* (i.e., ‘leaking’) has less relevance for the overall severity of the phenotype than overexpression of maternally silenced genes in UPD or other genetic factors in the paternally duplicated region. These findings suggest investigating UPD-specific pathophysiology in future studies.

In theory, IPD should present phenotypically like UPD (see Introduction and Fig. 1). Our finding that as a group, individuals with IPD have higher scores on various scales, i.e., presents clinically less severe, compared with UPD, and may therefore seem puzzling. This difference likely reflects frequent mosaicsm (~30%; genetic defect only in a subset of cells) in IPD [17]. This study did not systematically collect information on mosaicism. Future studies should investigate the impact of mosaicism on the phenotype of this genotype. As a working hypothesis, we may consider that IPD is composed of two subgroups (1) individuals with IPD without mosaicism (expected to present like UPD) and (2) individuals with IPD and mosaicism with a less severe phenotype.

We found that individuals with missense variants (MutM) have generally higher scores than individuals with truncating variants (MutT). In line with in vitro work [19], this suggests that a notable fraction of MutM have expression of *UBE3A* with residual functionality that leads to a less severe phenotype compared with truncating variants that have no expression of *UBE3A* from the maternal copy. Recently emerging data suggest that the variant type influence the localization of UBE3A within the neurons (cytoplasmic vs. nuclear), which is presumably critical for resulting phenotype [32, 33]. Given the low number and broad age range of individuals with MutM in our cohort (*n* = 14), a further investigation of these relevant question is beyond the scope of this publication.

In summary, the analysis of non-deletion AS revealed a complex picture suggesting different degrees of clinical severity that can be plausibly related to differences in the genetic irregularities.

### Psychometric properties of clinical scales for AS

We found that the BSID-III and PLS-4 scales had overall good psychometric properties across the AS population—the individual datapoints populated a wide dynamic range of each scale, and showed no apparent flooring effects (Fig. 2 and Supplementary Fig. 3). However, for individuals with non-deletion AS the BSID-III had expected or observed ceiling effects in 25.3% of individuals (Supplementary Table 8), which renders the use in older individuals with non-deletion AS problematic. For the VABS-2 scales, the picture is mixed—some scales are well-behaved, whereas some scales capturing higher abilities, e.g., the *written communication* scale, show clear flooring effects (Supplementary Fig. 3). This finding is not surprising given that individuals with AS are generally not capable of writing and the instruments are insensitive to other forms of communication. Overall, we found several signs of construct validity of these scales: scales separated deletion and non-deletion AS in the expected order, increased with age and had an overall plausible factor structure (Supplementary Table 11).

### Clinical severity scale

The CSS, albeit not a validated instrument, has good overall genotype discrimination; in particular, it is the only scale that captured differences between Del1 and Del2. Thus, the scale seems to capture variance in the AS population well and further development is recommended. There is room to improve the scale in several aspects. Currently there is one global CSS score derived as the sum of all items; however, some items have four levels of severity, while others have up to six levels, and consequently, items are weighted differently. The information content of different items may be investigated using e.g., item response theory to refine the list of items. Furthermore, it may be useful to group items into treatment-sensitive (current seizures, current abilities) and immutable (e.g., age of seizure onset, age of walking) groups for potential use as a response measure for treatments.

### Implications for future clinical trials and care

Our findings highlight the importance of taking genotype information into account in the clinical care and in clinical studies in patients with AS. Furthermore, we find that BSID-III (cognitive, communication, and motor domains), PLS-4 (communication domains) and some domains of the VABS-2 (communication, daily living skills, socialization, and motor domains) that capture clinical features reasonably well, show differentiation between genetic subgroups with different levels of impairment, and therefore may be useful as endpoints in this population (Supplementary Table 10).

### Limitations

Despite the overall large sample size, the number of individuals in non-deletion AS subgroups was limited. The scales investigated cover a limited scope of symptoms that may not address the specific aspects of each AS subpopulation. For example, specific symptom domains such as food seeking which may be relevant for UPD have not been assessed. Furthermore, a detailed consideration of epilepsy is beyond the scope of this publication and will be presented elsewhere.

## Supplementary information

Supplemental Material
