# Supplementary material for: Angelman syndrome genotypes manifest varying degrees of clinical severity and developmental impairment
Source: Mol Psychiatry. 2020 Aug 13;26(7):3625–33. doi: 10.1038/s41380-020-0858-6 (PMC8505254; doi:10.1038/s41380-020-0858-6)
Supplement: Supplementary file 1 — Supplemental Material [file 41380_2020_858_MOESM1_ESM.pdf]

Angelman syndrome genotypes manifest varying degrees of clinical severity and developmental impairment

Supplementary Information

Supplementary Patients and Methods

Participants

Initially, 1007 datasets from 304 participants enrolled in the study were available for analysis (we refer to the data from one participant collected at one visit as a single dataset). Per study protocol, participants were seen approximately annually over eight years. The mean number of visits per participant was 2.9 (±1.9); deviation from the expected 9 visits per participant was due to later enrolment in the study, missing visits and dropouts (Supplementary Fig. 1A).

Datasets from participants that could not be assigned to one of five genetic sub-groups (MutM, MutT, IPD, UPD, Del1, Del2) were excluded from the analysis (72 datasets from 36 participants). This included those with deletions of unspecified size, and those with incomplete testing that did not permit genotype assignment (e.g. abnormal DNA methylation, negative for deletion, but no further studies). Three patients with UBE3A mutations that were synonymous (i.e coding the same amino acid) were excluded. Furthermore, we included only datasets with a participant age between 1 and 18 years, because there were few datasets available for analysis outside of this age range (46 datasets from 18 participants). We excluded individuals with atypical deletions since only eight datasets were available (five longer than Del1, three shorter than Del2). Final analyses were based on 848 datasets from 250 participants (127 females). Mean age at clinic visits was 82.4 ± 45.3 months (median 73.9, inter-quartile range 47.4 – 111.6, Supplementary Figure 1B).

A molecular diagnosis was established for each participant using standard diagnostic testing for AS, involving fluorescent in-situ hybridization (FISH), methylation assay analyses, chromosomal microarray, microsatellite marker analysis, or gene sequencing. Supplementary Table 2 provides an overview of resulting diagnostic groupings of datasets entering the final analyses. 671 datasets were complete, i.e., data from all 19 scales were available. A table detailing the number of available assessments per scale can be found in the Supplementary Material (Supplementary Table 3). Since statistical modeling was carried out separately for each scale, we did not exclude any subjects based on missing values.

Clinical Scales

We analyzed data from the Bayley Scales of Infant Development, Third edition (BSID-III), the Vineland Adaptive Behavior Scales, Second edition (VABS-2), the Preschool Language Scale, Fourth edition (PSL-4) (all distributed by Pearson Education Inc., London, www.pearsonclinical.com), and the Clinical Severity Scale (CSS), a scoring tool developed specifically for the ASNHS. All assessments were carried out by trained personnel (physicians and licensed psychologists). The study protocol and tests performed were identical across all sites.

CSS is a severity scale created by the principal investigators at the main sites enrolling patients into the ASNHS and comprises a set of questions about symptoms typical for AS. The CSS has not been published previously and is reported here for the first time. It encompasses 11 assessments of severity: age of onset of epilepsy, current seizure frequency, number of seizure medications currently used,

current somatic growth (weight), current head growth, age of independent sitting, age of independent ambulation, presence/severity of scoliosis, verbal communication capabilities, nonverbal communication capabilities, and mean developmental age. Each assessment is scored on a 4- to 6-point Likert scale. We analyzed the CSS total score (sum of scores from all items). A detailed description of the CSS can be found in the Supplementary material (Supplementary Table 4).

BSID-III (1) is an interactive, play-based developmental assessment encompassing five subscales for fine motor, gross motor, receptive communication, expressive communication, and cognitive development. The BSID-III is normed for typically developing children up to the age of 42 months but is frequently employed to assess individuals with intellectual disability across a broader age span. It has been used to assess individuals with AS of all ages (2).

For some patients, when the psychologist expected or observed ceiling effects in BSID-III, the BSID-III was replaced by other scales (Supplementary Table 8).

VABS-2 (3,4) assesses adaptive behavior and is validated for a wide age range from birth to adulthood. It encompasses eleven subscales for expressive communication, receptive communication, written communication, fine and gross motor development, daily personal living, daily domestic, daily community living, social-interpersonal, social-play-leisure, and social coping abilities. The parent interview form was employed.

PLS-4 (5) is a an interactive assessment of language skills in children below 7 years of age, encompassing two subscales for receptive and expressive communication. The PLS-4 and the early items of the expressive and receptive communication domains of the BSID-III share high degree of overlap.

To facilitate interpretation, we assigned all subscales to functional domains. The assignment can be found in Supplementary Table 3.

Data analysis

All analyses were carried out within a linear mixed-effects model framework (LMM) using *R* (www.r-project.org) and the *nlme* library (6). A LMM, rather than a fixed-effects regression model, allows and accounts for correlations between repeated measurements from individuals, which makes it a powerful and flexible framework for the analysis of longitudinal data (7,8).

Since the scales analyzed were designed to capture child development, we expected all scores to be age-dependent. There are different data transformations that account for this dependence as provided in the scoring manuals, such as standard scores or developmental quotient tables. However, these tables are derived from typically developing (TD) individuals. Since our main interest was not a comparison between TD individuals and individuals with AS, but rather a description of the AS population including a comparison of different AS subgroups, we did not use TD-based age normalizations. TD-based age normalizations would have interfered with our analyses by introducing flooring effects and would not have accounted for age-dependence in the AS population given the fundamentally different developmental trajectories between individuals with AS and TD individuals (2,9). Therefore, we analyzed raw scores and accounted for age effects within our models, as described below. For the BSID-III scales, we additionally computed growth scale scores according to the manual, see Supplementary material.

We fit a LMM to the raw scores of each subscale and the CSS sum score. We modeled random intercepts per participant (to account for repeated measurements) and per study site (random intercept for each of the six centers of the study, to capture possible experimenter-induced covariance between participants seen at the same site). As fixed effects, we specified a third-order mean-centered orthogonal

polylogarithmic function of age (i.e., a third-order orthogonal polynomial of $\log_2$(age) – mean($\log_2$(age))). We chose this parameterization as a trade-off between model complexity and flexibility (in particular, to enable the models to capture non-linear developmental trajectories, such as plateauing, across the broad age range analyzed) following visual inspection of the data. Visual inspection was assisted by summary curves using locally estimated scatterplot smoothing (LOESS, Cleveland and Devlin, 1988) (see Figure 1, Supplementary Figs. 2, 3). We chose orthogonal linear, quadratic, and cubic terms for the LMM, such that no collinearity was introduced to the model.

First, we tested for differences between participants with (Del1, Del2) and without (MutT, MutM, IPD, UPD) deletions. For each scale, we compared a model using only age but no genotype information (M1) to a model with additional information about the presence or absence of a deletion and the interaction of the presence or absence of a deletion with age (i.e. with the polylogarithmic function of age, see above) (M2). Since differences between deletion and non-deletion participants have been found in several previous studies, we expected this model to fit the data significantly better than the data without genotype information for all scales. We then separated the dataset into deletion and non-deletion participants and further compared subgroups within them. We tested whether introducing diagnostic information concerning the class of deletion (Del1, Del2) and subtype of non-deletion (MutM, MutT, IPD, UPD) would significantly improve the models. All models were fit using the maximum likelihood (ML) method and were compared using likelihood ratio tests (LRT). The LRT compares the likelihood of the measured data, given a particular (full) model, with the likelihood for a nested (reduced) model. To assess whether the full model (containing one or several additional fixed or random effects compared with the reduced model) fits the data significantly better, the likelihood ratio for both models is subjected to a χ^2 test, since it asymptotically follows the χ^2 distribution under the null hypothesis (11).

When the best model contained the full diagnostic information for the non-deletion group, we performed pair-wise post-hoc comparisons between genotypes. In principle, post-hoc comparisons in a LMM can be carried out by extracting t-values and degrees of freedom (DF) from the model, but DF and p-value estimation is controversial (12). Therefore, we carried out post-hoc comparisons using likelihood ratio test model comparisons, by refitting the models with full genotype information and comparing them to models without genotype information, filtering out one genotype at a time from the dataset. We adjusted the p-values obtained in these post-hoc comparisons using the Benjamini-Hochberg method (13). This method adjusts p-values such that the expected rate of false positive results after adjustment is equal to the specified false discovery rate (FDR, e.g. 0.05).

We used the coefficients of the “best model” for each scale (i.e. the level of genotype detail as found in the analyses reported in Supplementary Table 5 and 6, and Table 1) to predict values at the sample mean ± standard deviation (std) of log-age (3.2, 5.8, 10.7 years) to generate a summarizing visualization of genotype differences (reported in Fig. 3, Supplementary Fig. 4). Furthermore, to investigate possible structure in the inter-individual variability across scales, we performed a factor analysis. To this end, we z-transformed (i.e., subtract mean and divide by standard deviation) the age-corrected data for each clinical scale. The z-transform was performed separately for individuals with and without deletions to ensure the co-variance structure was not driven by group differences between genotypes. We then subjected the normalized data to a factor analysis. Factors were computed using a maximum likelihood algorithm and oblimin rotation. Four factors were extracted, following the result of a Horn-parallel analysis.

We quantified the stability of the scales using intra-class correlation coefficients (ICC) based on the “best model” (see above). Since visits are spaced apart 1 year or more, the ICC values can be considered an upper bound for test-retest reliability, which is normally derived from measurements performed with much shorter intervals.

Supplementary Tables

Study	N	Symptoms	Results	Comment
Moncla et al., 1999 (14)	40	1.microcephaly, 2.ambulation delay, 3.age of onset of seizures, 4.seizure frequency	All symptoms more severe in deletion than in non-deletion	
Lossie et al., 2001 (15)	104	1.somatic growth delay, 2.gross motor impairment, 3.age of onset of seizures, 4.microcephaly	Symptom 1-3 more severe in deletion than in non-deletion; symptom 4 more severe in Mut and deletion than in IPD and UPD	
Peters et al., 2004 (9)	20	Global development	Tended to be lower in deletion than in non-deletion	Analyses not controlled for age, no inferential statistics reported because of small sample
Varela et al., 2004 (16)	58	1.absent speech, 2.independent sitting, 3.swallowing disorders, 4.hypotonia, 5.microcephaly 6.seizures	Symptoms 1 and 2 more severe in Del1 than in Del2; symptoms 3-6 more severe in deletion than in UPD.	49 participants with deletions, only 9 with UPD
Sahoo et al., 2006 (17)	22	1.cognitive delay, 2.language impairment, 3.autism diagnosis	Symptoms 1-2 more severe in Del1 than in Del2; autism diagnosis more frequent in Del1 than in Del2	Only participants with deletions
Gentile et al., 2010 (2)	92	1.cognitive skills, 2.gross and fine motor skills, 3.receptive language skills	All skills higher in non-deletion than in deletion; no differences found between Del1 and Del2	This study used a subset of our participant sample and part of the same assessment measures.
Luk and Lo, 2016 (18)	52	1. epilepsy, 2. microcephaly, 3. sleep problems	Epilepsy and microcephaly more common and sleep problems less common in deletion compared to non-deletion AS	
Shaaya et al., 2016 (19)	85	epilepsy	Epilepsy is higher for deletion compared to non-deletion AS	No formal statistical comparison
Bindels-de Heus et al., 2020 (20)	100	1. epilepsy (age of onset, types, and other related parameters), 2. growth parameters, 3. development (cognitive, motor, communication, using BSID-III)	More severe phenotype for deletion compared to non-deletion for epilepsy and developmental parameters	

Supplementary Table 1. *Review of developmental and clinical differences between AS genotypes reported in previous studies.*

Genotype	# Participants (Datasets)	Fraction of genotypes
Del1	69 (233)	27.6%
Del2	102 (352)	40.8%
Del1&2	171 (585)	68.4%
UPD	28 (80)	11.2%
IPD	21 (91)	8.4%
Mut	30 (92)	12.0%
MutM	14 (43)	5.6%
MutT	16 (49)	6.4%
Non-del	79 (236)	31.6%
All	250 (848)	100.0%

Supplementary Table 2. *Number of participants and dataset (in parentheses) analyzed by genotype. Right column provides percentages of the total number of participants analyzed.*

Scale	Datasets	Participants	Domain
BSID-III cognitive	781	244	cognitive
BSID-III receptive comm.	780	244	communication
BSID-III expressive comm.	781	244	communication
BSID-III fine motor	779	245	motor
BSID-III gross motor	791	247	motor
VABS receptive comm.	823	249	communication
VABS expressive comm.	823	249	communication
VABS written comm.	813	246	communication
VABS daily personal	822	249	daily living
VABS daily domestic	822	249	daily living
VABS daily community	822	249	daily living
VABS social interpersonal	822	249	social
VABS social play leisure	822	249	social
VABS social coping	822	249	social
VABS gross motor	764	244	motor
VABS fine motor	764	244	motor
PSL auditory	801	247	communication
PSL expressive	801	247	communication
Clinical Severity Scale	789	241	clinical

Supplementary Table 3. *Number of data-points and individual participants by scale.*

Severity Score			
	**Manifestation**	**Score**	**Definition**
Seizures	Age of onset of seizures	0	Never had seizures
		1	≥30 mos
		2	18mos to <30 mos
		3	12mos to <18mos
		4	6mos to <12mos
		5	<6 mos
	Epilepsy/Seizures at this visit	0	Absent
		1	<monthly
		2	< weekly to monthly seizures
		3	Weekly
		4	More than weekly
		5	Infantile spasms
	Number of seizure Medications	0	None
		1	1
		2	2
		3	3
		4	4
		5	≥5 OR requiring ketogenic diet OR vagal nerve stimulator
Growth	Somatic growth at this visit	0	No growth failure
		1	decrease in weight >1SD (10%≤w≤25%)
		2	decrease in weight >2SD (3%≤w≤10)
		3	decrease in weight >3SD
		4	decrease in weight >4SD
	Head growth	0	>25%
		1	11 -25%
		2	2-10%
		3	<2 %
Motor	Independent sitting by exam	0	Sits alone acquired ≤8months
		1	Sit with delayed acquisition (9 to 17 months)
		2	Sit with delayed acquisition (18 to 30 months)
		3	Sit with delayed acquisition >30 months
		4	Lost
		5	Never acquired
	Ambulation by exam	0	Acquired <18 months/ Ataxic gait
		1	18ms≤walks alone≤30ms (walks alone 18-30 months)
		2	Walks alone >30 months
		3	Walks with assistance >30 months
		4	Lost
		5	Never acquired

Scoliosis	Scoliosis	0	None or never had radiographs
	(assessed radiographically)	1	1^0 to $<20^0$
		2	20^0 to $<40^0$
		3	40^0 to $<60^0$
		4	$\geq 60^0$
		5	Surgery
Performance	Verbal Language	0	phrase speech
		1	>10 single words
		2	up to 10 single words
		3	no words, babbling (vowels/consonants strung together)
		4	grunts & non-word utterances (vowels only)
		5	No vocalization
	Nonverbal Language	0	Communication device (PECS, Alphasmart, etc.)
		1	uses signs and gestures (unprompted)
		2	uses signs and gestures (prompted)
		3	no signs or gestures
	Mean developmental age	0	Appropriate for age to 0.5 SD below mean
		1	0.6 – 1.5 SD below mean
		2	1.6 – 2.5 SD below mean
		3	2.6 – 3.5 SD below mean
		4	3.6 – 4.5 SD below mean
		5	> 4.5 SD below mean

Supplementary Table 4. *Items of the clinical severity scale (CSS).*

**Scale**	χ^2	***p***	***genotype fixed effect***	t_{MAIN}	**Domain**
BSID-III cognitive	192.45	**<.001**	14.3	14.76	cognitive
BSID-III receptive comm.	225.4	**<.001**	9.26	15.18	communication
BSID-III expressive comm.	128.14	**<.001**	5.79	11.73	communication
BSID-III fine motor	230.84	**<.001**	10.2	15.18	motor
BSID-III gross motor	107.24	**<.001**	6.94	10.08	motor
VABS receptive comm.	160.23	**<.001**	8.01	12.81	communication
VABS expressive comm.	124.37	**<.001**	7.8	11.87	communication
VABS written comm.	135.1	**<.001**	1.64	7.7	communication
VABS daily personal	167.22	**<.001**	11.56	11.37	daily living
VABS daily domestic	138.7	**<.001**	5.1	11.31	daily living
VABS daily community	117.29	**<.001**	4.34	9.84	daily living
VABS social interpersonal	139.24	**<.001**	6.37	11.84	social
VABS social play leisure	93.19	**<.001**	6.13	8.57	social
VABS social coping	134.97	**<.001**	5.05	10.48	social
VABS gross motor	82.68	**<.001**	11.58	9.19	motor
VABS fine motor	166.86	**<.001**	8.99	12.61	motor
PSL auditory	183.9	**<.001**	7.41	14.21	communication
PSL expressive	106.98	**<.001**	4.99	11.3	communication
Clinical Severity Scale	112.1	**<.001**	6.46	10.48	clinical

Supplementary Table 5. *Differences in clinical features between deletion and non-deletion AS. All statistics have been obtained in model-comparing likelihood ratio tests (df = 4);* χ^2*- and p-values effectively reflect main effect and deletion × age interaction together, t-values reflect main effect of deletion and thereby indicate the direction of the effect at mean log age. P-values have been corrected for multiple comparisons using FDR. Unadjusted p-values can be found in the Supplementary Table 9. The 'genotype fixed effect' column shows the main effect of genotype as estimated by the LMM models, in units of raw points. Positive values index better performance of non-deletion compared to deletion participants.*

Scale	χ^2	p	Deletion class fixed effect	t_{MAIN}	Domain
BSID-III cognitive	6.2	.314	1.1	1.4	cognitive
BSID-III receptive comm.	3.9	.528	1.0	1.8	communication
BSID-III expressive comm.	2.3	.681	0.4	1.0	communication
BSID-III fine motor	12.1	.088	0.5	1.3	motor
BSID-III gross motor	6.5	.314	1.2	1.9	motor
VABS receptive comm.	3.2	.614	1.2	1.5	communication
VABS expressive comm.	2.4	.681	0.3	0.5	communication
VABS written comm.	11.9	.088	0.4	1.7	communication
VABS daily personal	6.9	.314	1.3	1.4	daily living
VABS daily domestic	5.8	.338	0.5	1.4	daily living
VABS daily community	8.3	.258	0.5	1.0	daily living
VABS social interpersonal	6.3	.314	0.7	1.3	social
VABS social play leisure	4.9	.406	0.2	0.5	social
VABS social coping	3.1	.614	0.4	1.0	social
VABS gross motor	13.7	.080	3.1	2.3	motor
VABS fine motor	8.3	.258	0.9	1.6	motor
PSL auditory	7.4	.314	1.0	2.4	communication
PSL expressive	5.6	.338	0.7	1.9	communication
Clinical Severity Scale	20.7	**.007**	2.1	3.4	clinical

Supplementary Table 6. *Differences in clinical features between deletion class 1 and deletion class 2. T-values for main effect of deletion length (Del1 vs. Del2). P-values have been obtained in likelihood ratio tests (df = 4) and corrected for multiple comparisons using FDR. Unadjusted p-values can be found in the Supplementary Table 9. The 'genotype fixed effect' column indicates the main effect of genotype as estimated by the LMM models, in units of raw points. Positive values index better performance of Del2 participants.*

Scale	ICC	Domain
BSID-III cognitive	0.71	cognitive
BSID-III receptive comm.	0.65	communication
BSID-III expressive comm.	0.57	communication
BSID-III fine motor	0.68	motor
BSID-III gross motor	0.73	motor
VABS receptive comm.	0.61	communication
VABS expressive comm.	0.63	communication
VABS written comm.	0.42	communication
VABS daily personal	0.66	daily living
VABS daily domestic	0.53	daily living
VABS daily community	0.44	daily living
VABS social interpersonal	0.54	social
VABS social play leisure	0.46	social
VABS social coping	0.49	social
VABS gross motor	0.68	motor
VABS fine motor	0.66	motor
PSL auditory	0.65	communication
PSL expressive	0.58	communication
BSID-III cognitive growth score	0.70	cognitive
BSID-III receptive growth score	0.60	communication
BSID-III expressive growth score	0.56	communication
BSID-III fine growth score	0.66	motor
BSID-III gross growth score	0.72	motor
Clinical Severity Scale	0.78	clinical

Supplementary Table 7. *Stability of scales. Intra-class correlation coefficients (ICC) quantify the between subject variance relative to the total variance while accounting for variance related to the interaction of age (3rd order polynomial) and genotype and site. The visits are spaced 1 year and more apart. Thus, changes in scores reflect a combination of limited test-retest reliability and true change over time. The values can therefore be considered a lower bound for test-retest reliability.*

Genotype	# Participants (Datasets)	Ceiling in BSID-III # Participants (Datasets)	Fraction of visits
Del1	69 (233)	0 (0)	0.0%
Del2	102 (352)	1 (3)	1.0%
Del1&2	171 (585)	1 (3)	0.6%
UPD	28 (80)	3 (3)	10.7%
IPD	21 (91)	8 (13)	38.1%
Mut	30 (92)	9 (18)	30.0%
MutM	14 (43)	4 (8)	28.6%
MutT	16 (49)	5 (10)	31.3%
Non-del	79 (236)	20 (34)	25.3%
All	250 (848)	21 (37)	8.4%

Supplemental Table 8. *Left: Number of participants and dataset (in parentheses) analyzed by genotype (same as Supplemental Table 2). Right: Individuals and datasets (in parentheses) with "ceiling" of BSID-III (i.e. individuals and datasets where BSID-III was not performed since the capabilities were beyond what is captured by BSID-III as judged by the evaluator). The fraction of participants with ceiling differed significantly between deletion AS (Del1, Del2; 1 of 171 individuals with ceiling) and non-deletion AS (MutM, MutT, IPD, UPD; 20 of 79 individuals with ceiling) (Chi-square test;* $p = 5.6 \cdot 10^{-11}$*). The fraction of participants with ceiling did not differ significantly between MutM, MutT and IPD genotypes (Chi-square tests;* $p > 0.56$*). However, the fraction of participants with ceiling did differ significantly between UPD (3 of 28 individuals with ceiling) and the other non-deletion genotypes combined (MutM, MutT, IPD; 17 of 51 individuals with ceiling) (Chi-square tests;* $p = 0.027$*).*

Scale	Del vs NDel	Del1 vs Del2	IPD vs Mut	IPD vs UPD	UPD vs Mut	Domain
BSID-III cognitive	.000	.182	0.227	0.030	0.003	cognitive
BSID-III receptive comm.	.000	.417	0.859	0.033	0.001	communication
BSID-III expressive comm.	.000	.681	#NA	#NA	#NA	communication
BSID-III fine motor	.000	.016	0.584	0.102	0.008	motor
BSID-III gross motor	.000	.167	0.208	0.178	0.006	motor
VABS receptive comm.	.000	.519	#NA	#NA	#NA	communication
VABS expressive comm.	.000	.660	0.034	0.049	0.053	communication
VABS written comm.	.000	.018	0.164	0.005	0.000	communication
VABS daily personal	.000	.140	#NA	#NA	#NA	daily living
VABS daily domestic	.000	.215	0.182	0.000	0.001	daily living
VABS daily community	.000	.080	0.552	0.000	0.000	daily living
VABS social interpersonal	.000	.175	0.848	0.012	0.002	social
VABS social play leisure	.000	.299	0.959	0.004	0.001	social
VABS social coping	.000	.549	#NA	#NA	#NA	social
VABS gross motor	.000	.008	0.187	0.006	0.031	motor
VABS fine motor	.000	.081	0.585	0.031	0.000	motor
PSL auditory	.000	.118	0.060	0.053	0.000	communication
PSL expressive	.000	.231	0.021	0.382	0.000	communication
Clinical Severity Scale	.000	.000	0.141	0.010	0.049	clinical

Supplementary Table 9. *Unadjusted p-values from the pairwise post-hoc tests (see Tables 1, 2, Supplementary Tables 5, 6, 10 for FDR corrected p-values).*

**Scale**	χ^2	**p**	**t-value**	**genotype fixed effect**	**difference index [%]**	**Domain**
BSID-III cognitive	55.1	**<.001**	10.5	10.5	32.8	cognitive
BSID-III receptive comm.	56.4	**<.001**	7.2	7.2	26.6	communication
BSID-III expressive comm.	52.2	**<.001**	5.2	5.2	3.9	communication
BSID-III fine motor	67.9	**<.001**	7.5	7.5	30.7	motor
BSID-III gross motor	30.7	**<.001**	4.8	4.8	-37.5	motor
VABS receptive comm.	37.9	**<.001**	5.8	5.8	37.0	communication
VABS expressive comm.	41.2	**<.001**	5.6	5.6	24.1	communication
VABS written comm.	9.1	0.061	0.7	0.7	73.9	communication
VABS daily personal	37.2	**<.001**	8.9	8.9	15.1	daily living
VABS daily domestic	30.7	**<.001**	3.1	3.1	40.9	daily living
VABS daily community	18.0	**<.001**	3.0	3.0	40.2	daily living
VABS social interpersonal	33.0	**<.001**	5.3	5.3	25.6	social
VABS social play leisure	7.3	0.121	2.6	2.6	65.9	social
VABS social coping	42.9	**<.001**	4.5	4.5	12.9	social
VABS gross motor	28.0	**<.001**	7.2	7.2	-20.8	motor
VABS fine motor	38.3	**<.001**	6.8	6.8	28.1	motor
PSL auditory	43.6	**<.001**	5.3	5.3	32.9	communication
PSL expressive	37.6	**<.001**	4.3	4.3	-12.1	communication
Clinical Severity Scale	35.6	**<.001**	3.9	3.9	30.6	clinical

Supplementary Table 10. *Pair-wise post-hoc comparisons between UPD and Del2. P-values have been obtained through model-comparing likelihood-ratio tests and are corrected for multiple comparisons using FDR. The "genotype fixed effect" column indicates the magnitude of (numerical) group differences. Positive values index better performance for UPD. Unadjusted p-values can be found in the Supplementary Table 9. The difference index expresses the difference between UPD and MutT relative to the difference between DEL2 and MutT (((UPD-(MutT))/(DEL2-(MutT))*100%,* derived from age-corrected data, cf. Fig. 3)*. The 'genotype fixed effect' column indexes the fixed main effect of genotype (positive values index better performance for UPD participants), estimated by the LMM models, in units of raw points.*

**Scale**	**F1**	**F2**	**F3**	**F4**	**Domain**
BSID-III cognitive	0.6				cognitive
BSID-III receptive comm.	1.0				communication
BSID-III expressive comm.			0.9		communication
BSID-III fine motor	0.6				motor
BSID-III gross motor		0.9			motor
VABS receptive comm.	0.4				communication
VABS expressive comm.			0.5		communication
VABS written comm.				0.5	communication
VABS daily personal				0.5	daily living
VABS daily domestic				0.5	daily living
VABS daily community				0.7	daily living
VABS social interpersonal				0.7	social
VABS social play leisure				0.8	social
VABS social coping				0.6	social
VABS gross motor		0.9			motor
VABS fine motor				0.5	motor
PSL auditory	0.9				communication
PSL expressive			0.8		communication
Clinical Severity Scale		0.4			clinical

Supplementary Table 11. *Factor analysis: evidence for construct validity. The different clinical scales investigated test partially overlapping concepts. We hypothesized that scales designed to measure similar concepts such as e.g. gross motor symptoms or communication, would be highly correlated and map to one factor in a factor analysis. If this was the case it would provide evidence for the validity of the scales within the AS population. We therefore performed a factor analysis with all 19 age normalized and z-scored scales derived from the three factor model. The table shows the single highest rotated factor loading for each scale. The factor structure seems plausible: Factor 1 clusters scales capturing auditory receptive communication (but not the respective VABS scale), cognitive and fine motor abilities, Factor 2 clusters scales capturing gross motor skills and the CSS, which has several items related to motor skills as well, Factor 3 clusters scales capturing expressive communication, Factor 4 clusters scales capturing several higher-level skills measured by the VABS-III (daily living and social skills scales, written communication skills). Thus, there is a correspondence between the different scales that should measure similar domains, suggesting that meaningful variance is captured by theses scales in the AS population.*

Supplementary Figures

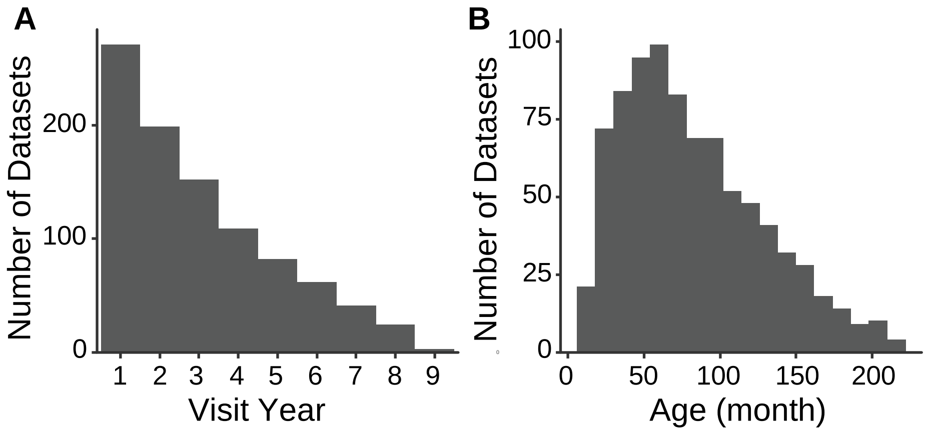

Supplementary Figure 1. *Number of datasets by visit year and participant age.*

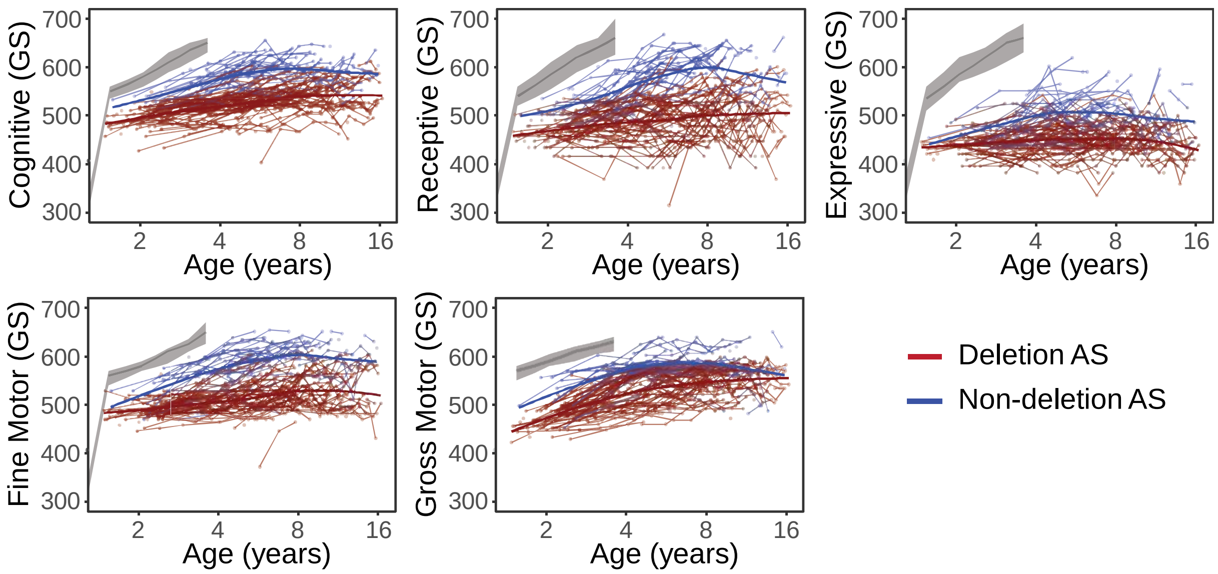

Supplementary Figure 2. *BSID-III growth scale scores for deletion and non-deletion as. Lines show spline fits (see BSID-III manual for details on growth-scale scores). Gray bands indicate median scores and inter-quartile ranges from a typically developing sample (data from the scale manuals, available for up to 3.5 years of age). Values from the same participant are connected by thin lines. Thick lines are the LOESS smoothing curves for deletion (blue) and non-deletion (red) participants.*

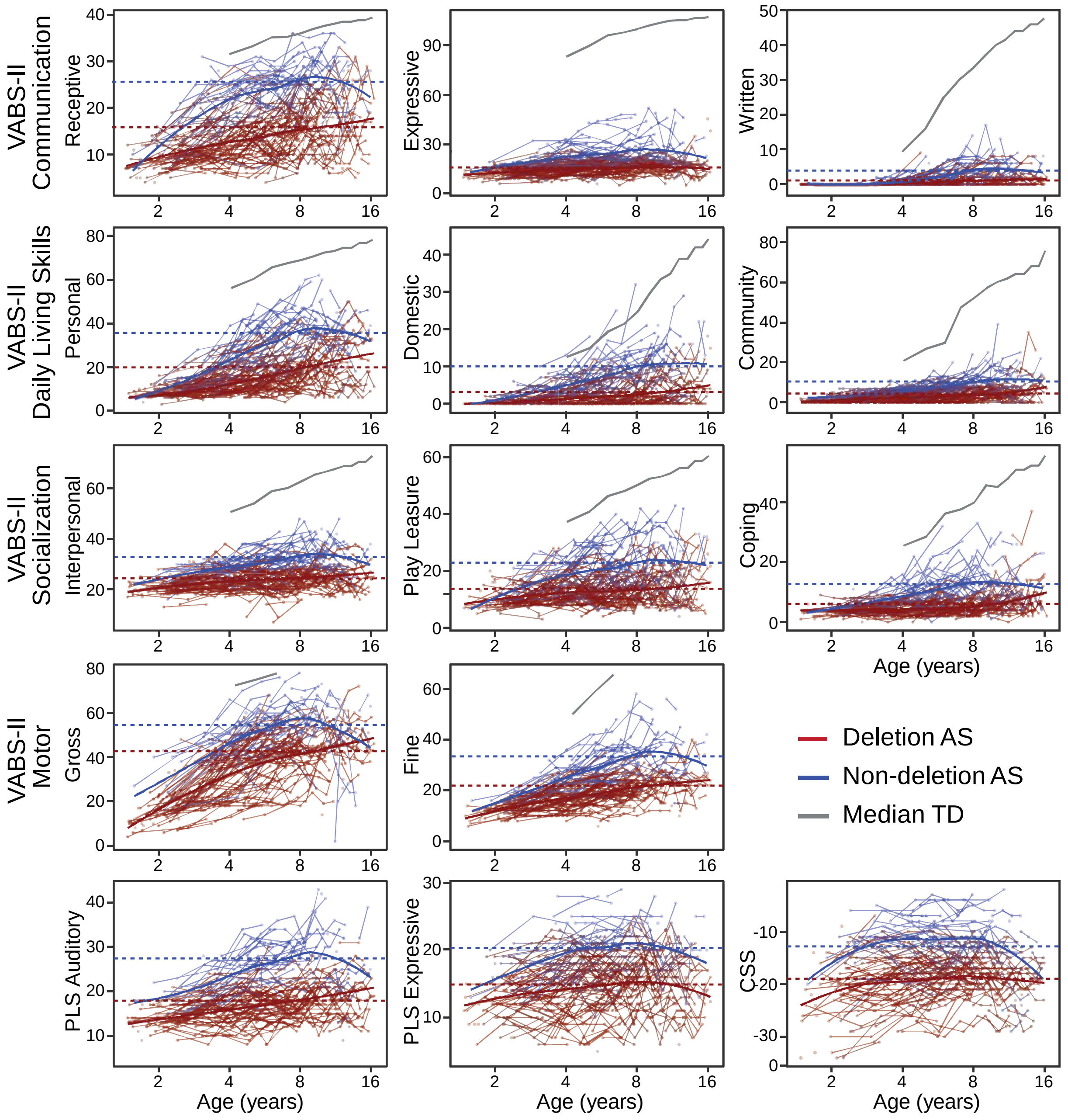

Supplementary Figure 3. *Raw data and norm scores (where available) for the VABS-II, PSL, and CSS scales as a function of age. Gray lines indicate median scores from a typically developing sample where available (VABS-II). Values from the same participant are connected by thin lines. Thick lines are the LOESS smoothing curves for deletion (blue) and non-deletion (red) participants. Note that these curves are cross-sectional data summaries, i.e., they do not account for within-subject longitudinal effects and are used for qualitative inspection of the developmental trajectory.*

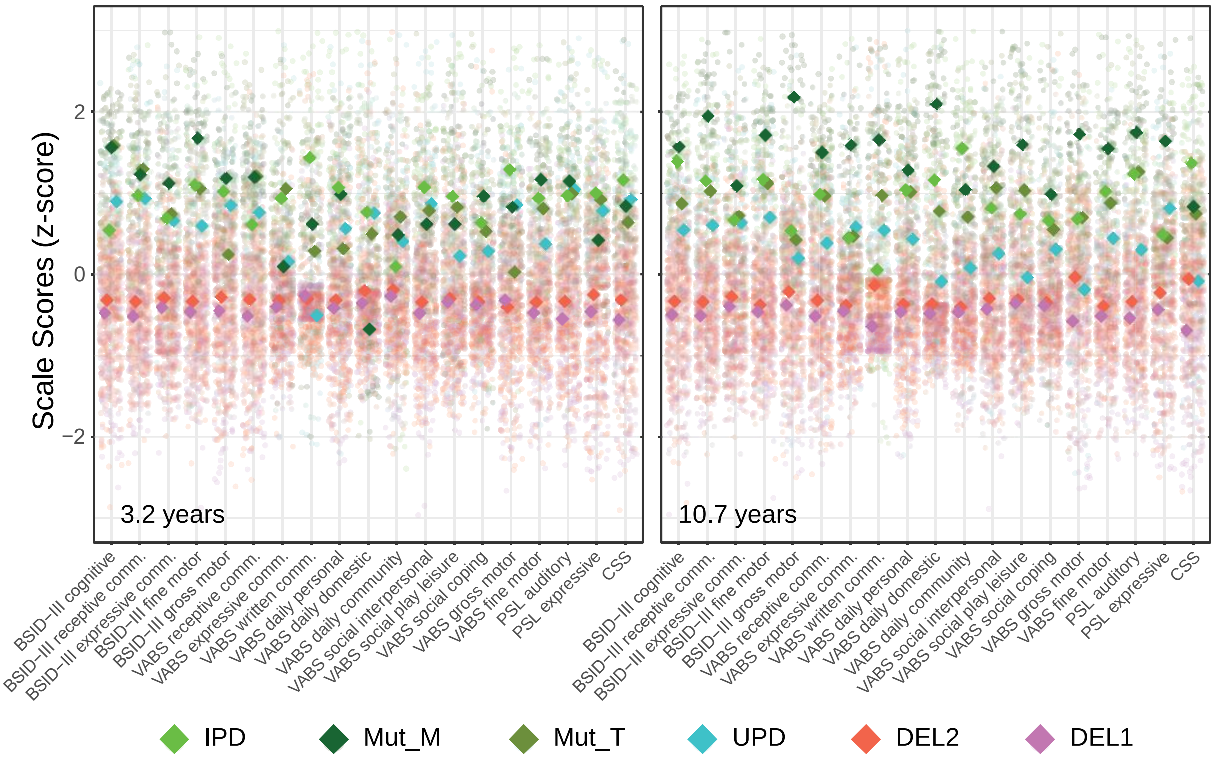

Supplementary Figure 4. *Comparisons of scores for different genotypes predicted for values at one standard deviation below and above the mean of log*$_2$ *of age (3.2 and 10.7 years). Z-standardized data from all participants and visits, derived from the respective 'winning' model. See Fig 3 for mean (5.2 years).*

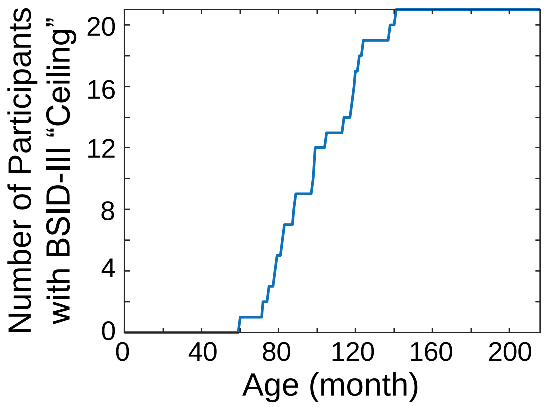

Supplementary Figure 5. *Cumulative number of individuals where BSID-III was not performed (due to expected ceiling) over age at assessment.*

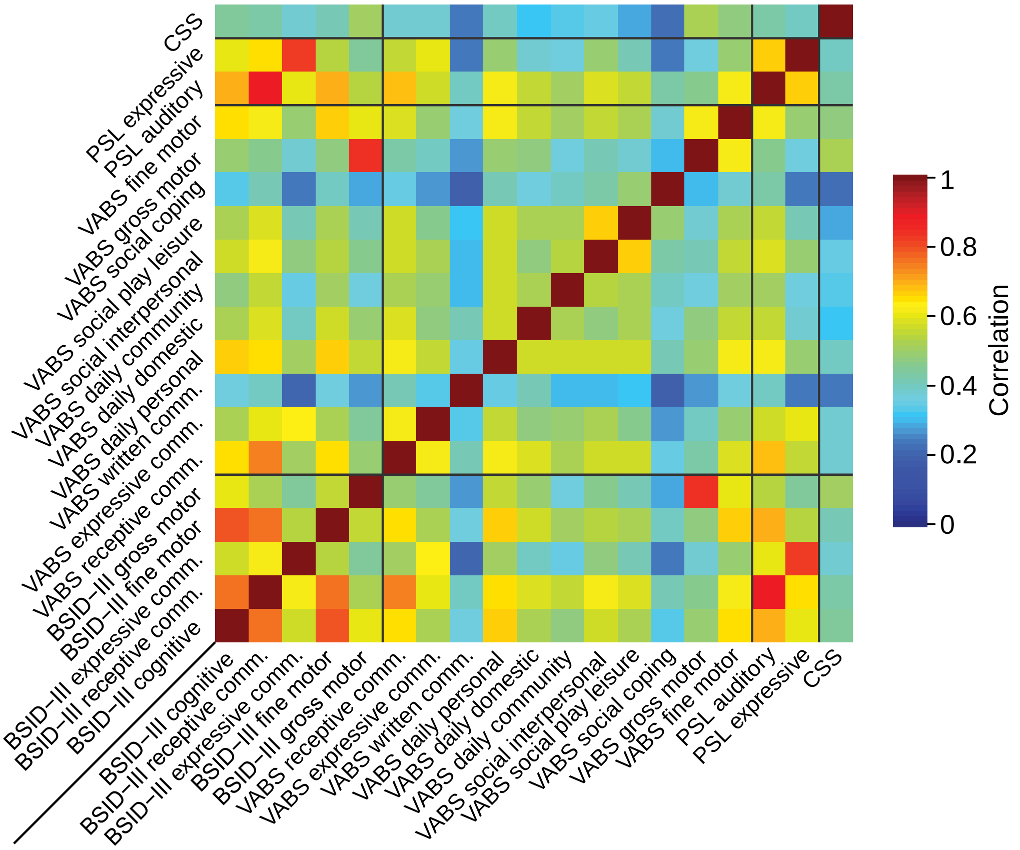

Supplementary Figure 6. *Correlations between scales. Data have been corrected for age differences and for differences between deletion and non-deletion (see Patients and Methods and Supplementary Table 11).*

Supplementary References

1. Bayley N (2006): *Bayley Scales of Infant and Toddler Development. San Antonio, TX: The Psychological Corporation*. Harcourt Assessment.
2. Gentile JK, Tan W-H, Horowitz LT, Bacino CA, Skinner SA, Barbieri-Welge R, *et al.* (2010): A neurodevelopmental survey of Angelman syndrome with genotype-phenotype correlations. *J Dev Behav Pediatr JDBP* 31: 592.
3. Sparrow SS, Cicchetti DV (1989): The Vineland Adaptive Behavior Scales.
4. Sparrow SS, Cicchetti DV, Balla DA (2005): Vineland adaptive behavior scales:(Vineland II), survey interview form/caregiver rating form. *Livonia MN Pearson Assess*.
5. Zimmerman IL, Castilleja NF (2005): The role of a language scale for infant and preschool assessment. *Ment Retard Dev Disabil Res Rev* 11: 238–246.
6. Pinheiro J, Bates D, DebRoy S, Sarkar D, Team RC (2017): Nlme: Linear and Nonlinear MixedEffects Models. R package version 3.1-128. 2016. *R Softw*.
7. Cnaan A, Laird NM, Slasor P (1997): Using the general linear mixed model to analyse unbalanced repeated measures and longitudinal data. *Stat Med* 16: 2349–2380.
8. Bates D, Mächler M, Bolker B, Walker S (2014): Fitting linear mixed-effects models using lme4. *ArXiv Prepr ArXiv14065823*.
9. Peters SU, Goddard-Finegold J, Beaudet AL, Madduri N, Turcich M, Bacino CA (2004): Cognitive and adaptive behavior profiles of children with Angelman syndrome. *Am J Med Genet A* 128: 110–113.
10. Cleveland WS, Devlin SJ (1988): Locally Weighted Regression: An Approach to Regression Analysis by Local Fitting. *J Am Stat Assoc* 83: 596–610.
11. Wilks SS (1938): The Large-Sample Distribution of the Likelihood Ratio for Testing Composite Hypotheses. *Ann Math Stat* 9: 60–62.
12. Vaida F, Blanchard S (2005): Conditional Akaike information for mixed-effects models. *Biometrika* 92: 351–370.
13. Ferreira JA, Zwinderman AH (2006): On the Benjamini–Hochberg method. *Ann Stat* 34: 1827–1849.
14. Moncla A, Malzac P, Voelckel M-A, Auquier P, Girardot L, Mattei M-G, *et al.* (1999): Phenotype–genotype correlation in 20 deletion and 20 non-deletion Angelman syndrome patients. *Eur J Hum Genet* 7: 131.
15. Lossie AC, Whitney MM, Amidon D, Dong HJ, Chen P, Theriaque D, *et al.* (2001): Distinct phenotypes distinguish the molecular classes of Angelman syndrome. *J Med Genet* 38: 834–845.
16. Varela MC, Kok F, Otto PA, Koiffmann CP (2004): Phenotypic variability in Angelman syndrome: comparison among different deletion classes and between deletion and UPD subjects. *Eur J Hum Genet* 12: 987.
17. Sahoo T, Peters SU, Madduri NS, Glaze DG, German JR, Bird LM, *et al.* (2006): Microarray based comparative genomic hybridization testing in deletion bearing patients with Angelman syndrome: genotype-phenotype correlations. *J Med Genet* 43: 512–516.
18. Luk HM, Lo IFM (2016): Angelman syndrome in Hong Kong Chinese: A 20 years' experience. *Eur J Med Genet* 59: 315–319.
19. Shaaya EA, Grocott OR, Laing O, Thibert RL (2016): Seizure treatment in Angelman syndrome: A case series from the Angelman Syndrome Clinic at Massachusetts General Hospital. *Epilepsy Behav* 60: 138–141.
20. Bindels-de Heus KGCB, Mous SE, ten Hooven-Radstaake M, van Iperen-Kolk BM, Navis C, Rietman AB, *et al.* (2020): An overview of health issues and development in a large clinical cohort of children with Angelman syndrome. *Am J Med Genet A* 182: 53–63.
